# Phenomenological Fingerprints of Four Meditations: Differential State Changes in Affect, Mind-Wandering, Meta-Cognition, and Interoception Before and After Daily Practice Across 9 Months of Training

**DOI:** 10.1007/s12671-016-0594-9

**Published:** 2016-08-19

**Authors:** Bethany E. Kok, Tania Singer

**Affiliations:** Department of Social Neuroscience, Max Planck Institute for Human Cognitive and Brain Sciences, Stephanstraße 1A, 04103 Leipzig, Germany

**Keywords:** Meditation, Meta-cognitive awareness, Decentering, Interoception, Affect, Multilevel modeling

## Abstract

Despite increasing interest in the effects of mental training practices such as meditation, there is much ambiguity regarding whether and to what extent the various types of mental practice have differential effects on psychological change. To address this gap, we compare the effects of four common meditation practices on measures of state change in affect, mind-wandering, meta-cognition, and interoception. In the context of a 9-month mental training program called the ReSource Project, 229 mid-life adults (mean age 41) provided daily reports before and after meditation practice. Participants received training in the following three successive modules: the first module (presence) included breathing meditation and body scan, the second (affect) included loving-kindness meditation, and the third (perspective) included observing-thought meditation. Using multilevel modeling, we found that body scan led to the greatest state increase in interoceptive awareness and the greatest decrease in thought content, loving-kindness meditation led to the greatest increase in feelings of warmth and positive thoughts about others, and observing-thought meditation led to the greatest increase in meta-cognitive awareness. All practices, including breathing meditation, increased positivity of affect, energy, and present focus and decreased thought distraction. Complementary network analysis of intervariate relationships revealed distinct phenomenological clusters of psychological change congruent with the content of each practice. These findings together suggest that although different meditation practices may have common beneficial effects, each practice can also be characterized by a distinct short-term psychological fingerprint, the latter having important implications for the use of meditative practices in different intervention contexts and with different populations.

**Electronic supplementary material:**

The online version of this article (doi:10.1007/s12671-016-0594-9) contains supplementary material, which is available to authorized users.

## Introduction

In recent years, the potential effects of mental training on well-being, brain, health, and behavior have become a focus of both popular and scientific interest. A wide variety of training programs now exist that offer secularized meditation training, comprised of standardized protocols with instruction in a variety of contemplative practices (Kabat-Zinn 1990), often in combination with other forms of mental training such as cognitive-behavioral therapy (Fjorback et al. 2011). Mental training has promise, both as a potential treatment for mental disorders from schizophrenia (Johnson et al. 2011) to PTSD (Lang et al. 2012) to depression and anxiety (Strauss et al. 2014), among others, and as a method for improving quality of life in individuals not diagnosed with a disorder (Chiesa and Serretti 2009).

Initial research on the benefits of secularized meditation programs rested on comparisons to passive control groups or on comparing pre-meditation and post-meditation effects in the same participants with no control (Ospina et al. 2008). Such studies found significant effects on physical and mental health, attention, stress reduction, and even brain activity (Chiesa and Serretti 2010). Other studies focused on defining the subjective experience, or phenomenology, of meditation, sometimes linking phenomenological measures to objective indices of neural or physiological activity (Lutz and Thompson 2003).

Meditation, like sports, refers to many different types of activities that can vary greatly in content, focus, effort, complexity, and duration. Widely studied secular mental training programs like Mindfulness-Based Stress Reduction (MBSR) or Mindfulness-Based Cognitive Therapy (MBCT) combine many different mental practices, making it impossible to isolate the effects of any one specific practice (Kabat-Zinn 1990; Williams et al. 2014). Furthermore, meditation is commonly performed in a specific context which can have effects independent of practice content. When meditative practices are compared to active control groups rather than waitlist controls or pre-post comparisons without a control group, findings of specific effects for meditation often become smaller and more bounded (Feldman et al. 2010; Kuyken et al. 2015; MacCoon et al. 2012; Obasi et al. 2013). Similarly, due to the intensive measurement requirements of phenomenological research, the vast majority of phenomenological studies focus only on the phenomenological space of one meditative practice, without direct comparison to others (for an exception, see Louchakova-Schwartz (2013)).

Four commonly studied meditative practices are breathing meditation, body scan, loving-kindness meditation, and observing-thought meditation. These practices are drawn mostly from the Buddhist contemplative traditions, although many other traditions employ similar exercises. Focusing on the breath is a meditation aimed at stabilizing attention and the mind; practitioners learn to direct attention to the breath in an intentional way, to monitor the direction of attention and detect when the mind wanders, and to return attention to the breath when mind-wandering is detected. As the practice teaches stable attention, an ability required for meditation, it is often taught to beginners yet remains central to the practices of many experienced meditators (Hart 1987; Sakyong 2003). In their review of the literature, Lutz et al. (2008) categorized focus on breath as a type of focused attention meditation and linked it to changes in attentional processing. For example, after practicing attentional focus meditation, experienced Tibetan Buddhist monks were able to perceive, as one stable percept, two dissimilar images presented to separate eyes; this effect did not occur after the same monks practiced compassion meditation (Carter et al. 2005).

The practice of body scan involves extending awareness to each individual part of the body in turn, typically starting at the head (Kabat-Zinn 1990). Practitioners focus on directing their attention exclusively toward the targeted body part and observing the sensations in that part. Body scan is an element of MBSR and is another kind of focused attention meditation, this time using different parts of the body as attentional objects. Body scan practice, in combination with focusing on the breath, improves interoceptive sensitivity and accuracy and also trains attentional control, as the meditator constantly monitors the object of attention and returns attention to the targeted body part if the mind wanders (Mirams et al. 2013).

Loving-kindness meditation is a practice that focuses on the cultivation of benevolence, love, and care toward others and the self (Salzberg 2005). Participants strengthen feelings of warmth and care through the visualization of a close loved person. Participants sequentially extend these feelings toward themselves, a close person, a neutral person, a person whom they dislike or have difficulties with, and finally toward strangers and human beings in general. Longitudinal studies comparing 8 weeks of loving-kindness meditation to a waitlist control have found that meditators increase in trait positive emotions (Fredrickson et al. 2008; Kok et al. 2013) and feelings of closeness to others (Kok et al. 2013) but do not change in negative emotions. There is also evidence that loving-kindness meditation induces neurological and physiological changes; 1 week of training in combined loving-kindness meditation and compassion increased neural activity in networks associated with positive affect and affiliation, relative to an active memory control group (Klimecki et al. 2013, 2014; Singer and Klimecki 2014).

Observing-thought meditation teaches “decentering,” a meta-cognitive process allowing thoughts to arise and fall without identifying with or becoming absorbed in their content or emotions. Learning to observe thoughts is an element of mindfulness meditation and is taught in courses including MBSR and MBCT (Fjorback and Walach 2012). Observing-thought meditation training cultivates meta-cognitive awareness of thoughts via two different meta-cognitive skills, taught sequentially. First, participants learned to categorize upcoming thoughts with labels such as “past,” “future,” “positive” or “negative,” and “self” or “other.” Then, participants learned to observe thoughts coming and going without reacting or engaging with the thoughts. Results of a recent empirical study suggest that training in observing-thought meditation has cognitive effects; compared to both body scan and mindful yoga, the observing-thought meditation practice of “sitting meditation” was associated with the greatest improvement in non-judging of thoughts (Sauer-Zavala et al. 2013). The opposite of decentering, persistent identification and enmeshment with thoughts, is known as rumination and is associated with a variety of negative mental health outcomes (Olatunji et al. 2013). Mindfulness meditation including an observing-thought component effectively reduced rumination relative to both relaxation training and measurement control groups (Jain et al. 2007).

These four meditative practices appear to have psychological and physiological effects when compared to waitlist control groups or to active non-meditative controls. There are also hints of practice-specific effects for breathing meditation and observing thoughts, compared to other meditative practices. Overall, however, while these four meditative practices have distinct conceptual bases and appear in theoretical work as distinct practices, there is no empirical evidence to directly differentiate them.

This study systematically investigates the common and differential state effects of these four meditation practices across dimensions of subjective experience commonly targeted by meditation, affect, mind-wandering, content of thoughts, and meta-cognitive and body awareness. Body scan and breathing meditation should most effectively increase feelings of presence and body awareness and decrease distraction by thoughts. Loving-kindness meditation should most effectively increase positivity of affect and feelings of warmth, as well as positive and other-focused thoughts. Finally, observing-thought meditation should not change the content of thought or decrease the amount of thoughts but should most effectively increase meta-cognitive awareness of thought contents and processes and decrease distraction by thoughts. In addition, we hypothesize that loving kindness will be more effective after 3 months of body scan and breathing practice. We also conducted exploratory network analyses of the interrelationships among the various outcome measures (Borsboom and Cramer 2013).

## Method

### Participants

Participants were recruited through flyers, radio and newspaper advertisements, and local news coverage in two major German cities. Potential participants attended one of multiple evening information sessions offered by the principal investigator (Singer), then indicated their interest in participating via a website. Potential participants were then sent a battery of screening questionnaires designed to identify individuals from vulnerable populations (underage, pregnant or nursing, suffering from mental or physical illness), individuals who would be unable to complete the behavioral or neurological measurement portions of the study, and individuals with previous meditation experience, all of whom were excluded from the present study. More details concerning participant recruitment, screening, and demographics in the ReSource Project are available in the online Supplementary material.

The sample at start of data collection included 80 participants in training cohort 1 (TC1), 81 participants in training cohort 2 (TC2), and 81 participants in training cohort 3 (TC3). The three training cohorts did not differ significantly in gender, age, or personality/mental health as assessed by a wide range of trait measures (listed in Singer et al. (2016), Appendix C2). Of the initial sample, 13 did not complete any meditation sessions using the online platform, thus providing no meditation data. Analyses are based on the following groups: TC1 78 participants, 58 % female, mean age 41.4 (minimum age = 20, maximum age = 55); TC2 78 participants, 59 % female, mean age 41.3 (minimum age = 21, maximum age = 55); and TC3 73 participants, 60 % female, mean age 40.7 (minimum age = 21, maximum age = 55). Additionally, by the start of the second module (affect for TC1, perspective for TC2), one participant provided no meditation data in TC1 and three provided no meditation data in of TC2. By the start of the third module (perspective for TC1, affect for TC2), four additional participants ceased to provide meditation data in TC1 and two participants ceased to provide meditation data in TC2. The total participant-level meditation data loss rate by the last module completed was thus 8.75 % for TC1, 10 % for TC2, and 10 % for TC3.

#### Procedure

Four different meditation practices were taught as part of the ReSource Project, a module-based 9-month secularized mental training program (Singer et al. 2016). The practices, different in content and goals, are matched in training context (e.g., length of retreats, amount of practice each week, length and structure of weekly group sessions, and a shared pool of teacher practitioners). As with most recent meditation research performed in the West, the training has been secularized. The first module, called presence, teaches two core practices; “breathing meditation” and “body scan” cultivate attention and interoceptive awareness. The second module, called affect, teaches “loving-kindness” meditation, with the purpose of creating positive, other-focused mental states and prosocial motivation. The third module, called perspective, teaches “observing-thought” meditation, in order to learn to identify and detach from the contents of thought and decrease reactivity to mental events. The affect and perspective modules also include near-daily dyadic meditations, the effects of which are discussed elsewhere (Kok and Singer, Contemplating the Other: Introducing the Contemplative Dyad and its effects on social closeness, motivation and personal disclosure over six months of mental training via a randomized clinical trial, under review). Participants underwent the same number of hours in retreats and teacher-led meditation training for all three modules. Teaching was conducted in teams to avoid confounding content with a particular teacher, although it was not possible to fully counterbalance teachers across practices to completely eliminate the potential effects of particularly effective teachers (Ospina et al. 2007). Each of the three modules of the ReSource Project serves as an active control for the other modules, allowing the assessment of practice-specific effects.

To explore sequence effects on training and create active control groups, participants were divided into three training cohorts, each of which experienced the modules in a different order. Training cohorts 1 and 2 began the study with a 3-day retreat led by experienced meditation teachers, where they started training breathing meditation and body scan (details about the retreats and content of the training program have been published in Singer et al. 2016). At the retreat, participants were also introduced to the ReSource data collection website, including guided meditation recordings and a series of online questions to be answered before and after meditating. For the next 13 weeks, participants were asked to practice breathing meditation for a minimum of 5 days a week and to conduct a body scan for a minimum of 5 days a week, using the guided meditation recordings and completing the pre-meditation and post-meditation questions before and after each practice. Guided meditations were available in 20-min (body scan) and 10-min (breathing meditation) lengths. In addition, participants attended weekly 2-h sessions with meditation teachers from the retreat.

**Fig. 1:**
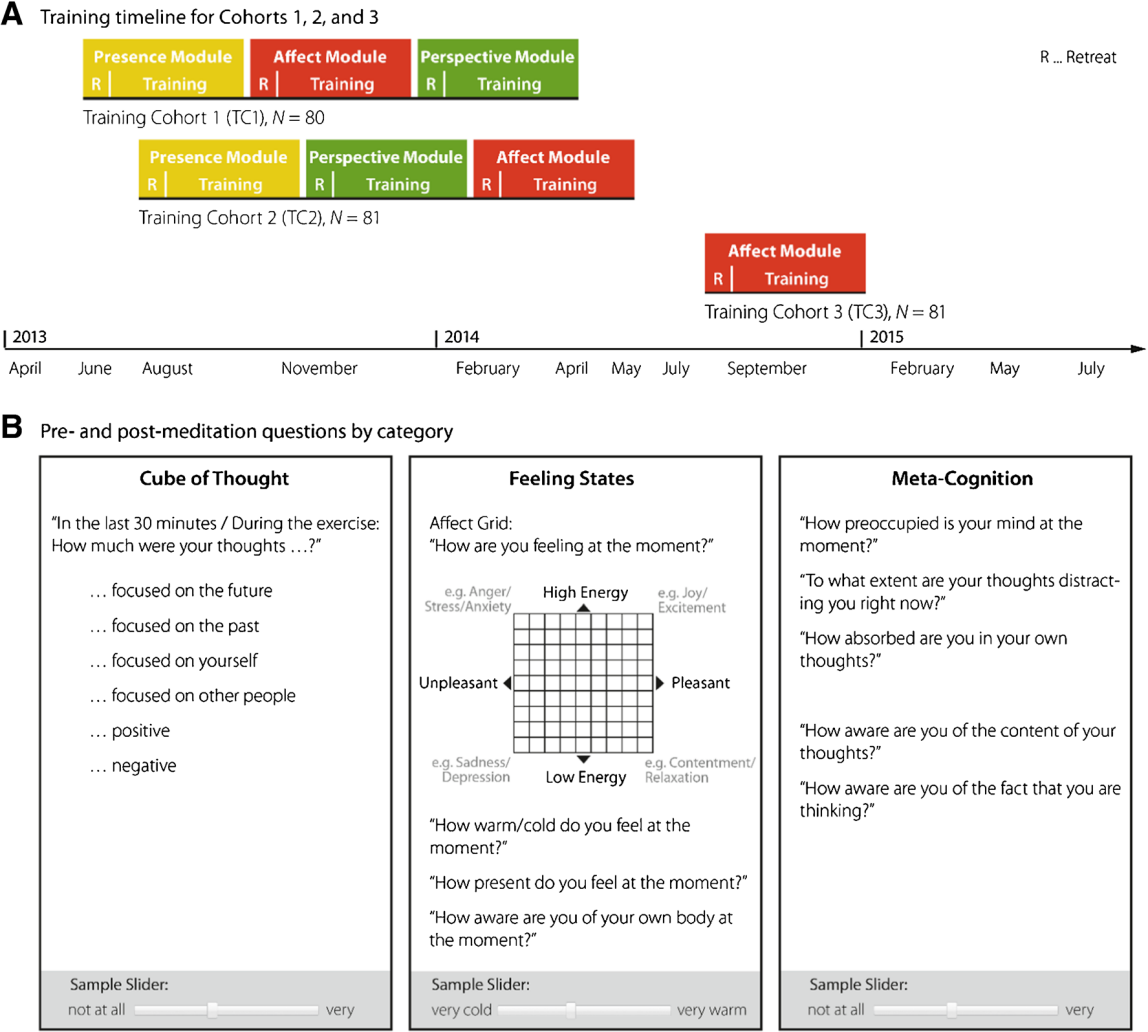
Study design, timeline, and day-to-day meditation measures. A fourth question block requesting that participants free-write about their mental state was also included in the design but is not discussed here. Section **A** adapted with permission from Singer et al. (2016), p. 36, Figure 4.1, with the timelines for control groups and long-term follow-ups removed

After 13 weeks of presence training (see also Fig. 1), participants in TC1 began 13 weeks of affect training in loving-kindness meditation and an emotion-focused dyadic exercise, starting with a 3-day retreat. Participants in TC2 began 13 weeks of perspective training in observing-thought meditation and a perspective-taking dyadic exercise, also starting with a 3-day retreat. For reasons of different format and content, the effects of the dyadic exercises will not be addressed here. As before, participants were asked to practice their core meditation (loving-kindness meditation for TC1 and observing-thought meditation for TC2) at least 5 days a week (in addition to a 2-h teacher-led group session each week), using guided meditation recordings available on the study website, and to complete the online questionnaire before and after practice. Participants also continued to attend their 2-h long weekly training sessions with meditation teachers, focusing on the new practice.

After the second 13-week training module was completed, participants in TC1 were assigned to the perspective module and participants in TC2 were assigned to the affect module, following the same pattern of retreat, meditation and dyadic activities, daily training and assessment, and weekly meetings described previously (see Fig. 1). Aside from the counterbalanced order of the affect and perspective modules and inevitable variations in season due to the staggered start times of the two training cohorts, participants’ experiences were intended to be identical across TC1 and TC2; all participants in both cohorts experienced the exact same combination of the same meditation practices taught by the same group of teachers, listened to the same text recorded on the meditation platform, and answered the same questions before and after daily practice.

Training cohort 3 attended a 3-day retreat structured similarly to the affect retreat for TC1 and TC2, where they were introduced to all core exercises of the affect module including loving-kindness meditation, and to the ReSource data collection website. Their practice expectations and assessment schedule were identical to TC1 and TC2, with the exception that they only practiced for one 13-week period.

At the end of the final 13-week period, training and assessment ended for all participants in all cohorts, although participants were still able to use the online meditation recordings and complete the questionnaires if they wished. Follow-up assessments were completed 4 and 10 months after the end of training. The results of those assessments will not be discussed here.

### Measures

The ReSource Project involved a wide range of assessments, with a full list available in Singer et al. (2016). Here, we analyze changes measured by a battery of quantitative pre-meditation and post-meditation questions.

In order to reduce demands on the participants’ time, questions were divided into four blocks, with two question blocks administered each day (see Fig. 1; note that the “open response” free-writing block is not shown in the figure). One block of questions (“feeling states”) was administered every day, while the others were presented in counterbalanced order across the weeks. The same question was asked before the meditation began and after it ended. Unless otherwise noted, answers were given using a continuous slider ranging from 0 (“not at all”) to 19 (“very much”).

The first question group was comprised of eight items, the six questions of the cube of thought, which assess the contents of thought, and two questions concerning attitude toward thoughts (Ruby et al. 2013). Participants indicated the extent that their thoughts were about the future, the past, the self, and others and how positive and negative these thoughts were. Participants then reported how much they had judged the thoughts (considering some thoughts good and some bad, for example) and how sure they were that they had accurately reported the content of their thoughts. These final two questions (“judging thoughts” and “sureness”) were not analyzed due to participant reports that those questions were difficult to understand. The pre-meditation questions concerned thoughts from the 30 min previous to answering the questions. The post-meditation questions concerned thoughts during the meditation.

The second question block assessed meta-cognition. Participants indicated the extent that in the 30 min before meditating (pre) and during the meditation (post), they felt “distracted by thoughts,” “occupied by thoughts,” and that their mind was “busy” with thoughts. Participants also indicated how aware they were of having thoughts and how aware they felt of the contents of their thoughts. The three questions concerning distraction (distracted, occupied, busy) were averaged to create a “thought distraction” score (mean daily *α* = 0.87, *SD* = 0.03). The two questions concerning thought awareness and thought content were averaged to create a “thought awareness” score (mean daily *α* = 0.77, *SD* = 0.06).

The third question block was comprised of 2 min of free-writing before and after the meditation, where participants were asked to record their thoughts and feelings as they occurred during the 2-min period. Analyses of these data are not yet complete.

The questions that were asked every day assessed affect, present-focused awareness, and interoception. They included an affect grid where participants reported valence and arousal just before and just after meditating, using a scale from 0 to 8 for each dimension (Russell et al. 1989). Participants also reported how warm they felt, how present they felt, and how aware of their body they felt.

In total, participants who followed the instructions to practice the core meditations 5 days a week would have completed each of the three question groups 20 times per 3-month period and completed the fourth daily group 60 times per 3-month period. Ultimately, we recorded approximately 66,390 measurement points representing over 11,000 h of meditation.

### Data Analyses

To test for within-person training effects, a three-level hierarchical linear model was fitted using the *nlme* package in R for each variable. Data was structured into measurement days (level 1), nested within practices (level 2), and nested within persons (level 3); comparisons between practices occur at level 2 and are within-person. Daily measurements were provided in groups of two, one before the meditation and one afterward. Tests of random effects (available in the online Supplementary material) revealed that, for all variables in TC1 and TC2, a three-level model was a better fit to the data than either a simple linear model or a two-level hierarchical linear model.

Each model included predictors representing type of meditation practice (*practice*, a categorical variable with four levels), whether the measurement took place before or after practice (*post*), and their interaction. State effects of practice were not moderated by a linear effect for the passage of time, and thus, the interactions of *time* with post and practice are not included in the final models described here; tests of the effect of time on the magnitude of state change are available in the Supplemental material. Details concerning the selection and coding of person-level and day-level covariates (including time), selection of random effects, contrast coding, the final model equations, and other model-specific information are available in the Supplementary material.

To test for between-person sequence effects, we fitted a two-level hierarchical linear model with time nested within participant for each variable. Each model included predictors representing training cohort (*training cohort*, a categorical variable with three levels), whether the measurement took place after practice (post), and their interaction. Details concerning the selection and coding of person-level and day-level covariates (including time), selection of random effects, contrast coding, the final model equations, and other model-specific information are available in the Supplementary material.

To explore potential changes in intervariate relationships, we utilized network analysis, a technique for graphically exploring relationships between multiple variables (Borsboom and Cramer 2013). Additional details are provided in the Supplementary material.

## Results

### Compliance

Participants were asked to practice the core meditation(s) of the current module at least five times per week, in addition to the weekly training session. Mean weekly meditation frequencies are shown in Table 1. For a detailed multilevel analysis of compliance rates by group and practice type over time, see section 10.2 of Singer et al. (2016). In general, compliance rates were higher for the presence module than for the other two modules.

**Table 1:** Number of meditation sessions per person per week

Meditation	TC1		TC2		TC3	
	Mean	SD	Mean	SD		
Breathing	4.89	1.19	4.52	1.25		
Body scan	4.38	1.20	4.34	1.24		
Loving kindness	3.89	1.14	3.38	1.30	4.05	1.57
Observing thoughts	3.57	1.22	3.69	1.24		

*SD* represents the between-person variance in mean compliance

### Within-Person Analyses

**Fig. 2:**
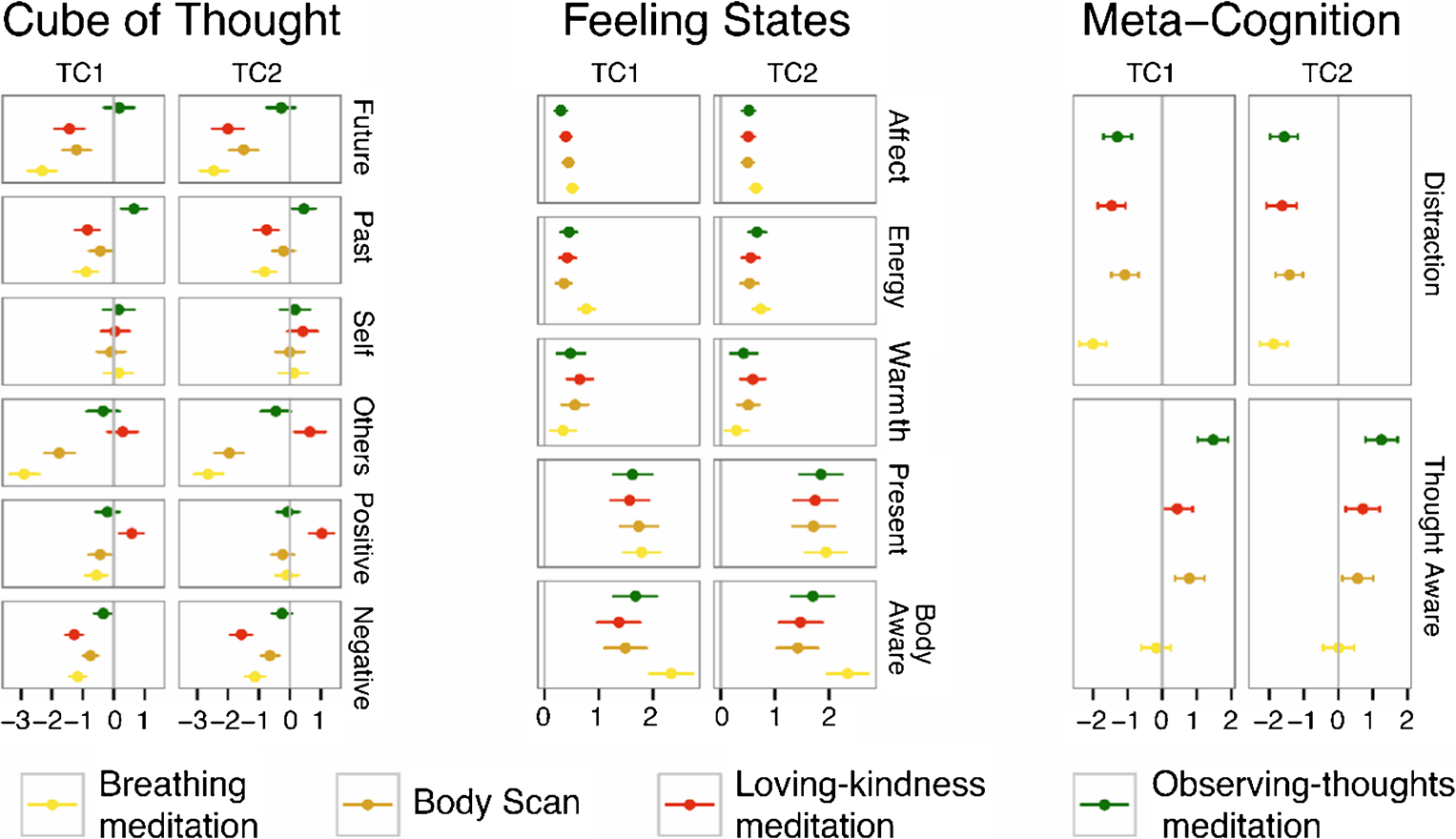
Estimates and 95 % confidence intervals for model-derived state changes in the four mental training practices of training cohorts 1 and 2

Table 2 provides the *p* values for the omnibus *F* tests, change estimates for each practice, and the contrasts among the practices as appropriate. Individual practice changes are also shown in Fig. 2. Information on the degrees of freedom, *F* values, and significance tests for all predictors are included in the Supplemental material. Models were also run without covariates (age, gender, media, time, weekend, Christmas), and the results showed a highly similar pattern of significance. These analyses are also included in the Supplemental material.

**Table 2:** Hierarchical linear model-derived state change estimates by training cohort

Characteristic	Training cohort 1					Training cohort 2				
	Breathing	Body scan	Loving kindness	Observing thoughts	*p* value	Breathing	Body scan	Loving kindness	Observing thoughts	*p* value
Future	−*1.20* ^a^	−*2.32*	−*1.44* ^a^	0.18	0	−*1.49* ^a^	−*2.46* ^b^	−*2.00* ^ab^	−0.28	0
Past	−*0.43*	−*0.90* ^a^	−*0.85* ^a^	*0.66*	0	−0.20	−*0.81* ^a^	−*0.76* ^a^	*0.45*	0
Self	−0.09^a^	0.16^a^	0.05^a^	0.16^a^	0.66	0.00^a^	0.13^a^	0.42^a^	0.17^a^	0.48
Others	−*1.76*	−*2.90*	0.29	−0.34	0	−*1.96*	−*2.64*	*0.65*	−0.45	0
Positive	−*0.44* ^a^	−*0.56* ^a^	*0.58*	−0.20^a^	0	−0.23^a^	−0.10^a^	*1.03*	−0.06^a^	0
Negative	−*0.76*	−*1.16* ^a^	−*1.28* ^a^	−*0.34*	0	−*0.64*	−*1.12*	−*1.57*	−0.25	0
Affect	*0.44* ^a^	*0.51* ^a^	*0.39* ^a^	*0.30*	0	*0.50* ^a^	*0.65*	*0.51* ^a^	*0.52* ^a^	0
Energy	*0.36* ^a^	*0.77*	*0.42* ^a^	*0.45* ^a^	0	*0.53* ^a^	*0.74* ^b^	*0.56* ^ac^	*0.67* ^bc^	0.01
Warmth	*0.56* ^ab^	*0.34* ^a^	*0.65* ^b^	*0.48* ^ab^	0	*0.51* ^a^	*0.29* ^b^	*0.59* ^a^	*0.42* ^ab^	0
Present	*1.74* ^a^	*1.79* ^a^	*1.57* ^a^	*1.62* ^a^	0.4	*1.71* ^a^	*1.94* ^a^	*1.74* ^a^	*1.85* ^a^	0.43
Body aware	*1.49* ^a^	*2.34*	*1.37* ^a^	*1.68* ^a^	0	*1.42* ^a^	*2.34*	*1.47* ^a^	*1.70* ^a^	0
Distraction	−*1.08* ^a^	−*2.00*	−*1.46* ^b^	−*1.29* ^ab^	0	−*1.42* ^a^	−*1.88* ^b^	−*1.64* ^ab^	−*1.57* ^ab^	0.06
Thought aware	*0.79* ^a^	−0.17	*0.44* ^a^	*1.47*	0	*0.56* ^a^	0.01	*0.71* ^a^	*1.25*	0

Change estimates significantly different from zero (*p* < 0.05) are indicated in ital. For each row within each training cohort, values sharing a superscript are not significantly different from one another (*p* ≥ 0.05)

The presence of two cohorts undergoing the same training program at different times means that TC2 can be interpreted as a replication of TC1. In light of this, correction for multiple testing was not used, but only statistically significant changes and practice differences that are shared across both cohorts will be interpreted.

While practicing breathing meditation did significantly increase present focus, this change was not greater than for the other three meditation types. Body awareness also increased significantly after breathing meditation practice but no more so than after loving-kindness meditation and observing-thought meditation and less than after body scan. Finally, there was a statistically significant decrease in the amount of thoughts encompassing three of the six descriptors of thought content (future, others, negative), and no significant increases in the amount of thought types for the other three thought content descriptors, indicating an overall decrease in thought quantity during breath-focused meditation.

**Fig. 3:**
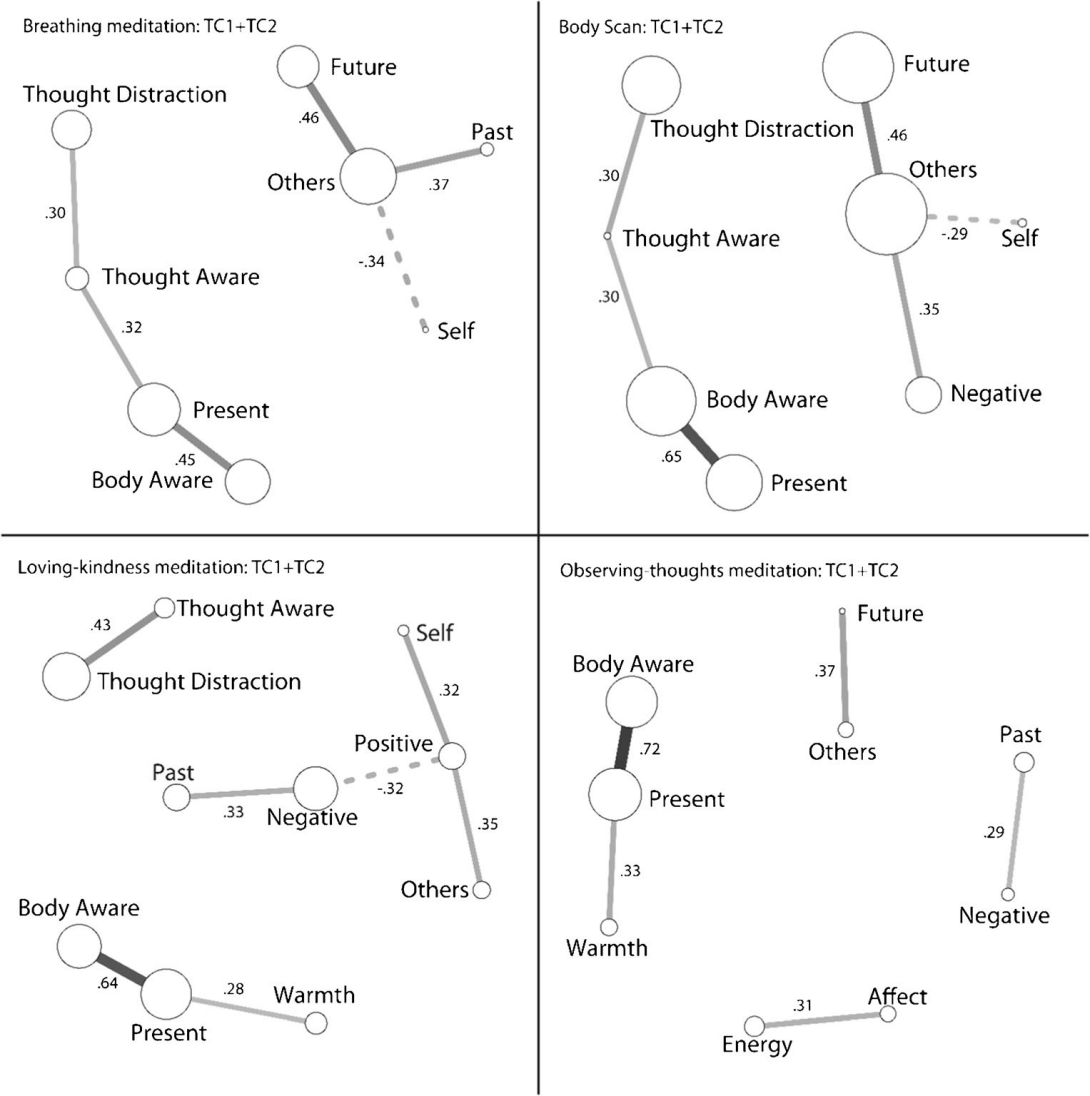
Partial correlation structures for each mental training practice combined across TC1 and TC2. Amount of change is represented by relative circle size and corresponds to the estimates in Table 2. Paths between *circles* represent the significant partial correlations, with *solid lines* representing positive and *dashed lines* representing negative correlations. Variables without significant correlations are not shown

As shown in Fig. 3, the phenomenological space for breathing meditation was defined by two clusters, one comprised of meta-cognition, interoceptive awareness, and present-moment focus and the other linking thoughts of others to future-oriented, non-self-focused thoughts.

Body scan showed the highest increase in body awareness of all practices studied; present focus also increased but no more than for other practices. Body scan was also associated with a statistically significant decrease in the amount of thoughts encompassing four of the six descriptors of thought content (future, past, others, negative), and no significant increases in the amount of thoughts for the other two descriptors of thought content, indicating an overall decrease in thought quantity during body scan meditation.

Consistent with similarities in training and goals for the two practices, the phenomenological space for body scan is very similar to that of breathing meditation, two clusters, one characterized by links between present focus, body awareness, thought awareness, and distraction by thoughts and one representing the structure of non-task-related thought (mind-wandering).

As anticipated, during loving-kindness meditation, participants reported the greatest increase in positively valenced thoughts and in fact, were the only group to show statistically significant positive change in that variable. Regarding other-focused thoughts, loving-kindness meditation again had the greatest increase in other-focused thoughts relative to the other groups, but this positive change was only significant for TC2. In TC1, this difference was mainly due to large decreases in other-focused thought in breathing meditation and body scan. Contrary to our hypothesis, while loving-kindness meditation was related to increases in subjective warmth, these changes were no greater than the warmth changes that occurred during the breathing and observing-thought meditations, although they were higher than the change that occurred in body scan. Finally, all four practices demonstrated statistically significant increases in positivity of affect, but the increase associated with loving-kindness meditation was not reliably larger than in the other practices as had been hypothesized.

In loving-kindness meditation, changes in the phenomenological space are divided into three clusters. Awareness of thoughts and thought distraction are now distinct from awareness of the body and the present moment, the latter now linking to feelings of warmth. Finally, the thought content cluster is consistent with the goal of loving-kindness meditation linking positive thoughts to self- and other-oriented thoughts and negatively to a cluster of negatively valenced, past-related thoughts.

As anticipated, observing-thought meditation led to the largest statistically significant increase in thought awareness of all four practices. Observing thoughts also significantly decreased distraction by thoughts; however, this change was not statistically different from the change in thought distraction caused by breathing meditation or loving-kindness meditation. In TC1, body scan practice caused participants to be even less distracted by thoughts than observing-thought meditation.

Observing thoughts led to four small, narrowly defined clusters. One cluster now relates affect and energy; the second past-negative thoughts; the third future-other related thoughts; and the last, similar to loving-kindness meditation, links body awareness, present focus, and warmth.

All four practices showed significant increases in positivity of affect, present focus, ability to avoid being distracted by thoughts, energy, and body awareness. “Selflessness” is an additional pursuit often associated with meditation. However, in both TC1 and TC2, there was no significant change in the amount of self-related thoughts for any of the four practices.

### Between-Person Analyses 

**Fig. 4:**
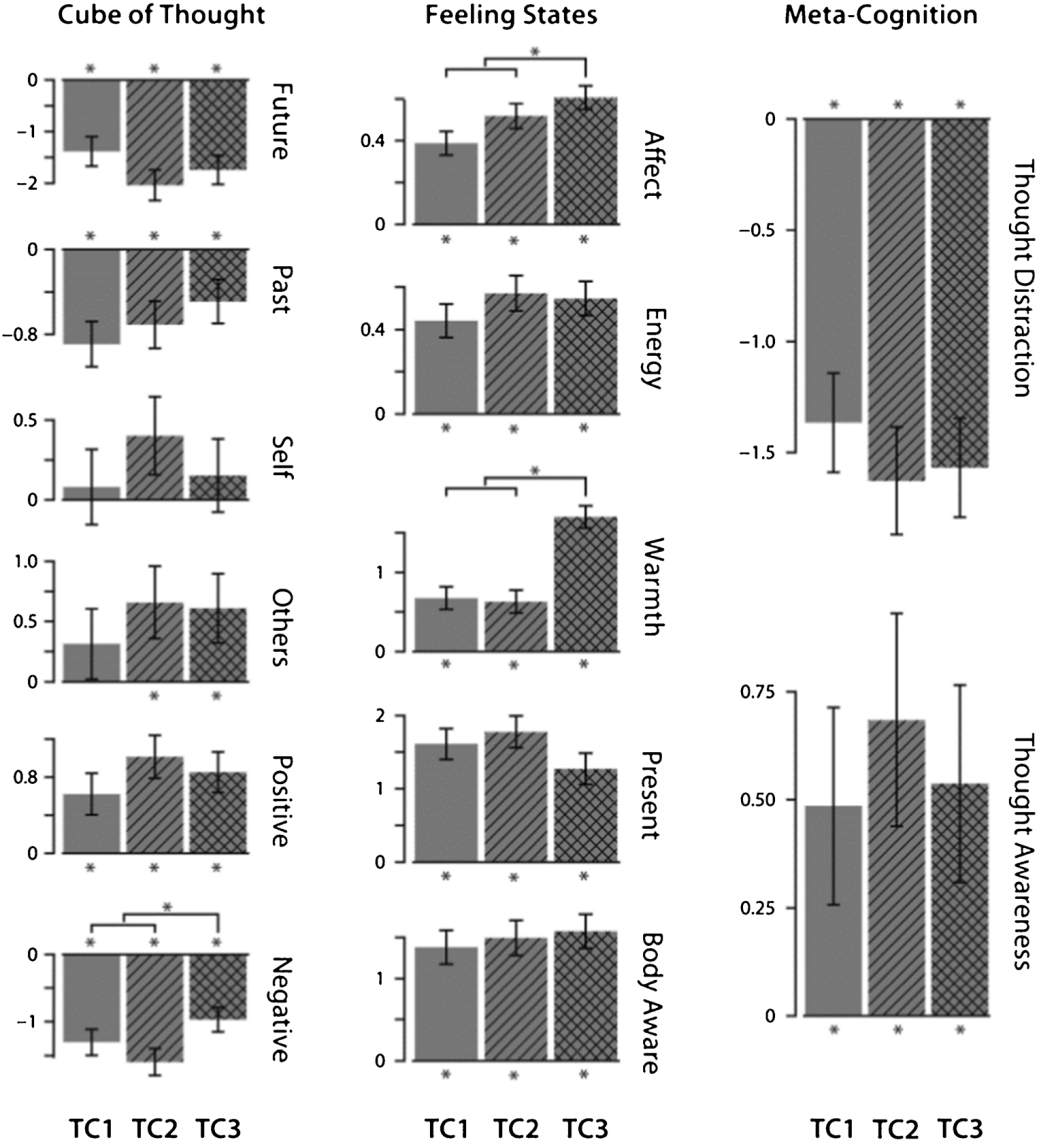
Model-derived state change estimates for loving-kindness meditation for training cohorts 1, 2, and 3. The *error bars* represent the person-level standard errors. The *stars* represent change significantly different from zero

Figure 4 shows the differences between the training cohorts in the effects of loving-kindness meditation, reflecting a significant effect for interaction of *group* and *practice*. Information on the significance tests for all coefficients is included in the Supplemental material. Models were also run without covariates (*age*, *gender*, *media*, *time*, *weekend*), and the results showed a highly similar pattern of significance. These analyses are also included in the Supplemental material.

For the majority of variables, there was no difference between groups in the amount of change occurring due to loving-kindness meditation. Critically, even in the absence of 3 months of attention training, loving-kindness meditation still increased present focus, body awareness, thought awareness, and ability to disengage from distracting thoughts just as much as in other training cohorts, despite the fact that these changes are more closely associated with presence training than with loving-kindness meditation.

Some unanticipated differences between cohorts did appear. Participants who did not receive preparatory presence training, but did the affect module immediately, showed a significantly smaller decrease in negative thoughts during meditation compared to the other groups. These participants also showed the highest increase in positivity of affect and the greatest increase in subjective warmth.

**Fig. 5:**
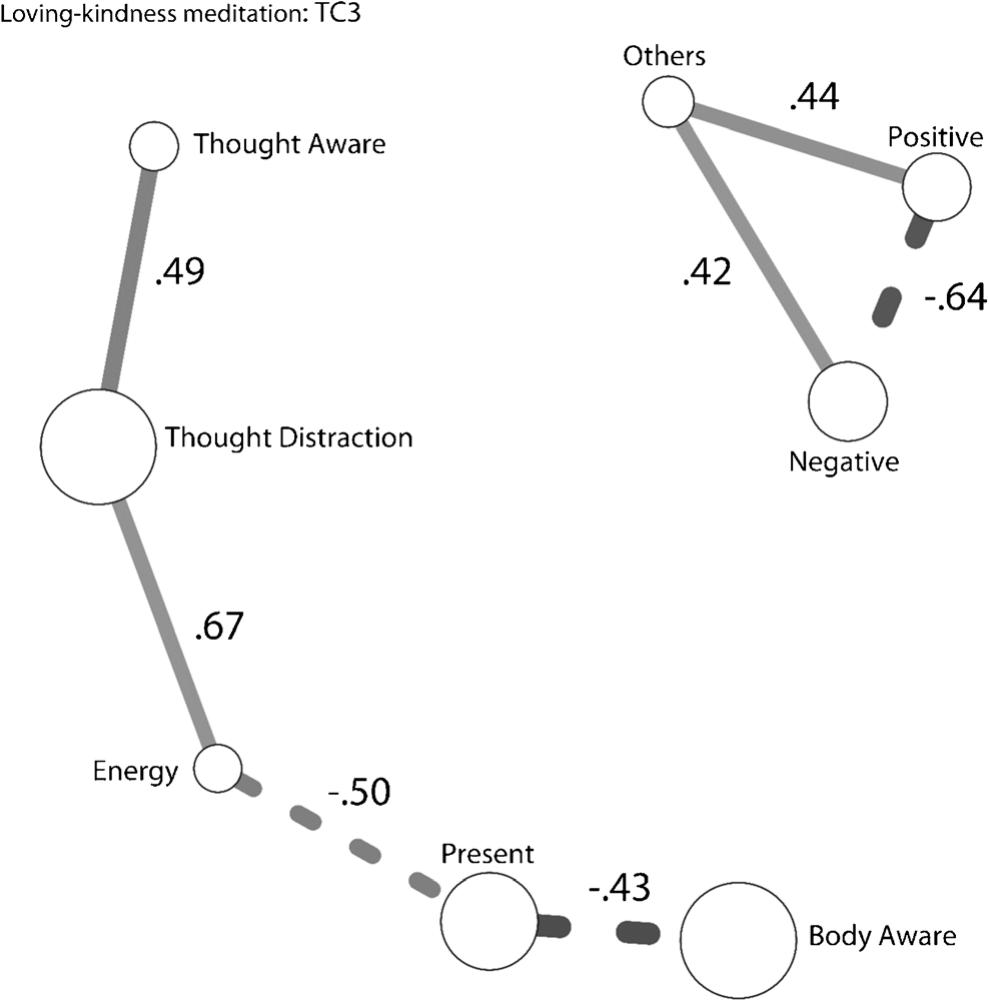
Partial correlation structures for each TC3. Amount of change is represented by relative circle size. Paths between *circles* represent the significant partial correlations, with *solid lines* representing positive and *dashed lines* representing negative correlations. Variables without significant correlations are not shown

The pattern of intervariate relationships for loving-kindness meditation without the presence module (shown in Fig. 5) differed somewhat from the pattern for loving-kindness meditation with presence training. While thoughts of others were still associated with positively valenced thoughts and decreased negatively valenced thoughts, the relationship between thought awareness and present-focus was different. In the original two cohorts, there was no link between thought awareness and present focus in loving-kindness meditation, although they were positively linked in body scan and breathing meditations. Here, they are negatively linked through changes in energy, suggesting that in the absence of presence training, participants found it difficult to sustain awareness of their thoughts and the present moment simultaneously.

## Discussion

The purpose of the present work was to compare the differential psychological effects of daily practice of four different types of meditation included in the Resource Project, a 9-month long longitudinal study of mental training (Singer et al. 2016). We specifically focused on identifying patterns of training-related change in experienced affect, thought content, meta-cognition, and body awareness. Training consisted of three 13-week modules (presence, affect, perspective), each comprised of two core practices that targeted attentional, socio-affective, and socio-cognitive functions, respectively. Two of the core practices were dyadic in nature, and their effects will be discussed elsewhere. The other four were single-person meditations, breathing meditation and body scan (presence), loving-kindness meditation (affect), and observing-thought meditation (perspective). Training modules were matched in the time required, the learning environment, the structure and length of retreats, the frequency of practice, the availability of support through the online platform and a smartphone app, and teacher investment. We expected differential effects of these types of mental practices on subjective outcome measures assessed before and after daily practice of a given meditation.

As hypothesized, each of the four mental practices showed unique “mental fingerprints” of change. These fingerprints were expressed as differential training-related effects on self-reported measures of affect, cognition, and body and meta-cognitive awareness, as well as differences in the structure of the relationships among variables as illustrated by the network analyses.

Specifically, breathing meditation, trained during the presence module, decreased the overall number of thoughts and the tendency to be distracted by thoughts and increased present focus, interoceptive awareness, positivity of affect, warmth, and energy. The fingerprint of body scan, also trained during the presence module, was similar to that of breathing meditation. Participants decreased in overall number of thoughts and the tendency to be distracted by thoughts and increased present focus, positivity of affect, and energy. Body scan led to the highest increase in body awareness of all four practices. Network analyses of breathing meditation and body scan revealed two main clusters, the first linking awareness of thought and distraction by thoughts to body awareness and present focus and the second linking increased thoughts about others and thoughts about the future to decreased thoughts about the self. This suggests that participants may have used body focus and present-moment focus as a way of coping with distraction by thoughts. Such a strategy is congruent with the training goal to increase awareness of all present-moment experience, including bodily sensations and thoughts. The second cluster could be interpreted as “mind-wandering,” as attention is diverted from the self (and the breathing or bodily sensations that are the target of the meditations) toward others and potential future or past events.

Loving-kindness meditation led to expected increases in positively valenced thoughts, a change not observed for the other three practices. Other-focused thoughts also increased relative to the other practices, but only training cohort 1 showed a statistically significant absolute increase in other-focused thought. Feelings of subjective warmth and positivity of affect also increased but no more than for the other practices. As with all other practices, participants also increased present focus, interoceptive awareness, and energy. This mental pattern maintained both for loving-kindness meditation learned in sequence with other modules and loving-kindness meditation taught without other modules. Loving-kindness meditation without other modules, however, was more effective at enhancing warmth and positivity of affect.

Follow-up network analyses further differentiated loving-kindness meditation from other practices. Changes in meta-cognition were uncoupled from changes in interoceptive awareness and present focus, suggesting a shift from cultivating general attention to domain-specific attention. Furthermore, body awareness and present focus were not only positively related to each other but also to warmth. Finally, positively valenced thoughts were related to thinking about self and others, and this thought pattern was negatively associated with negatively valenced, past-focused thoughts. This last “thought cluster” is consistent with the instructions of loving-kindness meditation to visualize loved others and extend good wishes of loving kindness and happiness to them (Salzberg 1995). It further suggests that the more participants think positive thoughts of self and others, the more ruminative thoughts (negative and past-focused) are suppressed. Interestingly, the uncoupling of meta-cognition and interoception in loving-kindness meditation occurred only when loving-kindness meditation was taught after the presence or perspective modules. When the affect module was taught in isolation, meta-cognition and interoception were positively associated, suggesting that this dissociation is not inherent to loving-kindness meditation but may result from approaching loving-kindness meditation with already established meditation skills.

Finally, observing-thought meditation, trained during the perspective module, led as expected to the highest increase in meta-cognitive awareness of thoughts. Participants were also less distracted by thoughts but no more so than for other practices. The goal of observing-thought meditation is to refine the ability to categorize and observe thoughts without reacting, as well as to cultivate awareness of the thought process. This is manifest in the network analysis of many small, separate clusters of conceptually linked variables, indicating a nuanced approach to thoughts similar to what is trained in MBCT (Fjorback et al. 2011; Strauss et al. 2014; Williams et al. 2014). The cluster of affect and energy represent the participant’s subjective state, while the past/negative cluster may reflect a common ruminative pattern and the future/other cluster has been linked to planning (Ruby et al. 2013). The body awareness and present-focus cluster is also conceptually consistent, although the inclusion of warmth is unexpected. Finally, thought awareness and thought distraction are uncoupled in observing-thought meditation, suggesting that participants become more proficient in distinguishing between meta-cognitive awareness of thoughts and being distracted by thoughts. As for the other practices, participants also increased in present focus, interoceptive awareness, positivity of affect, warmth, and energy. Interestingly, no practice significantly changed the amount of self-related thought experienced by participants.

In summary, the following effects uniquely defined each of the four core meditative practices: body scan led to the greatest increase in interoceptive awareness, loving-kindness meditation was best in increasing positively valenced and other-focused thought and was the only practice to positively link thoughts of self and others, and observing-thought meditation was most effective in increasing meta-cognitive awareness of thoughts and resulted in the highest number of distinguishable thought-content clusters. There were no unique effects for breathing meditation, which is used as a basic practice in many contemplative traditions.

The fingerprints observed for the meditative practices, both in individual variable changes and in distinct networks of related intervariate change, provide validation for the idea that the type of meditation matters. The choice of what type of meditation to engage in, or what to focus on within a particular type of meditation, has experiential consequences. Other studies have shown that repeated subjective experiences such as anxiety (Kubzansky et al. 2006) or positive affect (Fredrickson et al. 2008; Kok et al. 2013) can have significant and far-reaching consequences for life, health, and longevity. In such a context, meditation may prove a critical tool in helping to “shift” the tone of subjective experiences away from those known to be noxious and toward more salubrious states.

Our findings have a number of every day and clinical implications. By demonstrating distinct subjective “fingerprints” for four types of meditation taught in a shared context and assessed using a shared question pool, we provide clear evidence that some forms of meditation may be better suited to certain professions or patient populations than others. The fingerprints revealed here may help practitioners to choose a meditative practice that is the best “match” for the current needs of their client. In addition, the overlapping effects shared between practices also suggest that in some cases, the choice of meditation can be guided by individual preference without compromising the efficacy of the chosen practice.

Our findings also have implications for researchers who may wish to study the effects of repeated subjective experiences. Challenges in such research include attempting to reliably and regularly induce the desired subjective experience and finding an effective matched control. Additional analyses, reported in the Supplemental material, suggest that the magnitude of change is relatively consistent over time, indicating a lack of habituation. Furthermore, since the state effects of breathing meditation appear to be a subset of the effects found in the other three practices, pairing breathing meditation with body scan, loving-kindness meditation, or observing-thought meditation would allow the researcher to observe the long-term effects of repeatedly experiencing interoceptive awareness, positive other focus, or meta-cognitive awareness, respectively.

While practice-specific hypotheses were mostly confirmed for breathing meditation, body scan, loving-kindness meditation, and observing-thought meditation, there was also a substantial amount of un-hypothesized overlap between the different meditations. All practices left participants feeling happier, more energized, more present in the moment, more aware of their bodies, and better able to disengage from distracting thoughts.

Some of the shared or overlapping effects may be attributed to continuing practice in directing, re-focusing, and sustaining attention to a given object in the present moment, be it the breath, parts of the body, a mental image, or a mental event like a thought. Learning to focus attention and stay in the present moment, detect when attention has wandered, and return attention to the original target are fundamental to all contemplative practices (Kabat-Zinn 1990), and a muscle is exercised by all four mental training exercises studied here. The analogy may be to a runner learning the geography of a city; depending on where the runner goes, she will learn about a different neighborhood, but no matter where she runs, her muscular and cardiovascular system will show a similar pattern of development over time. In the same way, different meditative practices can simultaneously offer unique benefits while sharing a common underlying pattern of gains.

Other effects, such as the improvements in mood and energy, could be due to the pleasant effects of learning a new self-care skill. It could be also argued, since participants in training cohorts 1 and 2 received breathing meditation and body scan training before learning the other two practices, that these apparent “commonalities” might in actuality represent carryover effects from the previous module. We were, however, able to investigate such sequence effects; TC1 and TC2, where loving-kindness meditation was learned after the presence module, were compared to TC3, where loving-kindness meditation was taught without any prior training. As described above, we found few differences and many commonalities. In particular, the increases in present focus, interoceptive awareness, and energy, and decreases in distraction by thoughts, did not differ significantly across the affect modules in TC1, TC2, and TC3. This suggests that cultivating bodily awareness and present focus is a core common feature of our four meditative practices (or their context), as is feeling more positive and energized after contemplative practice of any kind.

We have repeatedly referred to the richness of this data due to the intensive measurement strategy of the project. While the consistent and extensive measurement of participants’ subjective states is a strength of this work, it retains the weaknesses common to subjective self-report measures. The project was designed to account for these weaknesses; asking participants to report their mental state in the moment eliminates retrospective biasing of responses, and recording answers using sliding scales rather than discrete values reduces the ability to repeat a particular response from memory. However, self-report remains only as valid as the participant’s own ability to introspect. Collecting many repeated measurements from each participant, and using multilevel modeling to identify overall trends, helps to separate the “signal” of real effects from the “noise” of error.

Similarly, the within-person nature of much of the ReSource Project design is a strength that allows inferences about intrapersonal change due to meditation. However, within-person designs are vulnerable to sequence effects. In the case of the two main training cohorts (TC1 and TC2) of the ReSource Project, the presence module was always taught first because it laid the foundation for the other two modules, affect and perspective. As practice compliance decreased over time, differences between the presence practices and the other two core practices (loving-kindness meditation in affect and observing-thought meditation in perspective) could in part be attributed to higher compliance rates in the presence module. Differences between breathing meditation and body scan, however, would not be affected by changes in compliance. Furthermore, the counterbalanced training sequence for the affect and perspective modules across TC1 and TC2 controls for the potential effects of compliance differences for those two modules.

Finally, participants in TC1 and TC2 underwent 9 months of meditation training, a much longer period than is typical in meditation research. A longer training period is useful for observing whether the effectiveness of the various practices changes over time. Training for three quarters of a year, however, means that the three modules were inevitably taught in noticeably different seasons, with accompanying differences in temperature, amount of sunlight, day length, etc. Seasonal effects could be potentially confounded with differences in training content. However, training in TC2 began 2 months after TC1. In combination with the counterbalanced order of the perspective and affect modules, the offset group design ensures that none of the training modules were taught twice in the same season. In addition, as shown in the Supplemental material, there were no differences in the magnitude of state changes for the two presence practices between TC1 and TC2, despite being taught in different seasons. Thus, we conclude that the effects of the training were not season-dependent.

Seasonal and compliance differences are confounding factors in the between-person design used to investigate sequence effects in the affect module across the three training cohorts. The affect modules taught in TC1, TC2, and TC3 differ in sequence, season, and compliance rates. Thus, any differences between the modules could potentially be due, in whole or in part, to non-training-related differences. It is all the more surprising, therefore, that almost no group differences were found; the affect module training thus appears to be robust against variation in seasons and compliance rates as observed in this study.

In conclusion, the unique fingerprints associated with different types of contemplative mental training reveal that meditation is not about engaging in indiscriminate mental effort; each practice appears to create a distinct mental environment, the long-term consequences of which are only beginning to be explored.

## Electronic Supplementary Material

Below is the link to the electronic supplementary material.

ESM 1 Additional recruitment, screening, and demographics information; model equations and tests for all model terms; alternative models. (PDF 1330 kb)
