# Supplementary material for: Phenomenological Fingerprints of Four Meditations: Differential State Changes in Affect, Mind-Wandering, Meta-Cognition, and Interoception Before and After Daily Practice Across 9 Months of Training
Source: Mindfulness (N Y). 2016 Aug 19;8(1):218–31. doi: 10.1007/s12671-016-0594-9 (PMC5241345; doi:10.1007/s12671-016-0594-9)
Supplement: Supplementary file 1 — Additional recruitment, screening, and demographics information; model equations and tests for all model terms; alternative models. (PDF 1330 kb) [file 12671_2016_594_MOESM1_ESM.pdf]

Phenomenological Fingerprints of Four Meditations: Differential state changes in affect, mind-wandering, meta-cognition and interoception before and after daily practice across nine months of training

Mindfulness

Bethany E. Kok* and Tania Singer

Department of Social Neuroscience, Max Planck Institute for Human Cognitive and Brain Sciences

*Correspondence concerning this article should be addressed to Bethany E. Kok, Department of Social Neuroscience, Max Planck Institute for Human Cognitive and Brain Sciences, Stephanstraße 1A, 04103 Leipzig, Germany (e-mail: bethkok@cbs.mpg.de)

Contents **Pages**

Recruitment and screening 3

Demographics 5

Description of final within-person model 6

Description of final between-person model 9

Network analysis 11

Tests of nested random effects for within-person models 12

Moderation of state change by time 14

Significance tests for all model terms for within-person models 16

Significance tests for all model terms for between-person model 19

Within-person model results without covariates 22

Effect of season on magnitude of state change in training 23

References 24

Recruitment and screening

The recruitment and screening procedure for the *ReSource Project* was a multi-step process in order to inform the participants in an appropriate manner, screen for eligibility, and ensure motivation for a large-scale one year longitudinal study, including extensive scientific testing.

Participants were recruited by various means, e.g., flyers, local newspaper articles and advertisements, TV and radio announcements, as well as flyers in public transportation systems in Leipzig. Participants for TC1, TC2, and RCC1vwere recruited in Winter 2012/2013 and for TC3 and RCC2 in Winter 2013/2014. (RCC was collected in two waves (RCC1 and RCC2) to avoid time-of-year confounds when interpreting the effects of TC3.)

First screening: Potential participants completed an online questionnaire to screen for demographic data, time constraints (training sessions, retreats) and physical and mental health factors

Exclusion criteria for the study were as follows:

- Not between 20 and 55 years old
- No computer access at home/no internet connection
- Not meeting our MRI safety house standards (no irremovable metal in the body, tattoos on the upper part of the body, permanent make-up, pregnancy or lactating mothers, obesity, diabetes, neurological disorders, head trauma with loss of consciousness, peripheral vascular diseases, peripheral arterial diseases, Reynaud’s diseases, involuntary motor disorders, epilepsy, insulin pumps, retainer, inner ear implants, pacemakers, drug pumps, cerebral water drainage)
- Regular spiritual practice in the last 2 years
- Regular meditation practice in the last 2 years, participation in meditation retreats
- Does not speak/understand German fluently
- Chronic pain
- Psychotherapy in the last 2 years
- Allergic to adhesive tape
- Smoking more than 5 cigarettes per week
- Drugs and alcohol abuse
- Diagnosed mental disorders (ok if recovered more than two years ago)
- Cortisol intake
- Medications (that affect central nervous system function, psychotropics, opiates, corticosteroids, medications for anxiety, depression, or other psychological problems)
- Studies or studied psychology

Those who passed the initial screening criteria then completed a battery of mental health questionnaires. Potential participants were excluded if they scored above a pre-determined cut-off on measures of a specific mental illness:

- Major (ICD-10) Depression Inventory[1], exclusion from mild to severe;
- Toronto Alexithymia Scale[2,3], exclusion if > 60;
- State-Trait-Angstinventar[4], exclusion if > 56;
- Patient Health Questionnaire-D[5];
- Prescreening question for the Structured Clinical Interview for DSM-IV for axis II personality disorders (SCID-II)[6], as a basis for an psychological interview;

Once past this screening, they received a second set of questionnaires that served as a basis for grouping participants into training and control cohorts via replacement randomization.

- IQ: Grundintelligenztest Skala 2 - Revision (CFT 20-R) ohne WS/ZF-R[7]
- Test d2 – Revision (Cognitive ability)[8]
- Ten Item Personality Inventory[9]
- Self-Compassion Scale[10]
- Perceived Stress Scale[11]
- Five Facet Mindfulness Questionnaire[12]
- Multidimensional Assessment of Interoceptive Awareness[13]

- Freiburger Beschwerdeliste (list of physical problems)[14]
- Mental Health Continuum Short Form[15]
- Interpersonal Reactivity Index[16]
- Compassion for Others scale [17]

Second screening: After passing the online screening and completing the questionnaires, participants were invited to an obligatory information evening at the institute and given a description of the time commitments and activities required by the study. After confirming their continued interest to take part in the study by e-mail, a face-to-face mental health diagnostic interview with a trained clinical psychologist was scheduled. The interview included:

- a computer assisted version of the Structured Clinical Interview for DSM-IV Axis-I disorders[6],
- the DIA-X [18]
- the German version of the SCID-II for Axis-II disorders [19,20].

Participants were excluded if they fulfilled the criteria for an Axis-I disorder within the past two years, if they at any point fulfilled the criteria for schizophrenia or other psychotic disorders, bipolar disorder or substance dependency, or if they fulfilled the criteria for an Axis-II disorder.

All successful participants gave written consent for their participation.

Text adapted from Chapter 7.1 Recruitment of Study Participants, pp.46-49[21]

Demographics

Start of study	TC1	TC2	TC3
N	80	81	81
Age Mean(95% CI)	41.29 (41.07/41.51)	41.19 (40.94/41.43)	40.44 (40.22/40.67)
% Female	58.75	59.26	60.49
% Married	41.77	38.46	29.49
% Unmarried and cohabiting	30.38	20.51	34.62
% Single	27.85	41.03	35.9
Provided any meditation state change data			
N	78	78	73
Age Mean(95% CI)	41.37 (39.38/43.37)	41.32 (39.13/43.52)	40.74 (38.63/42.85)
% Female	57.69	58.97	60.27
% Married	39.74	38.46	24.66
% Unmarried and cohabiting	29.49	19.23	30.14
% Single	28.21	38.46	28.77
Provided meditation state change data for all assigned modules			
N	73	73	
Age Mean(95% CI)	41.37 (39.25/43.49)	41.04 (38.79/43.39)	
Female	56.16	60.27	
% Married	38.36	36.99	
% Unmarried and cohabiting	30.14	17.81	
% Single	28.77	41.10	

Description of final within-person model

Practice-mean-centered time, whether the measurement took place on a weekend (*weekend*), whether the measurement took place over the two weeks between Christmas and New Years (*Christmas*), whether the measurement was done online or using the smartphone app (*Media*), participant gender, and participant age were included as covariates to improve model fit and enhance interpretability of results. Further tests of random effects resulted in keeping second- and third-level correlated random effects for intercept, *Post, time, Media* (meditation done via smartphone or web platform), and *weekend*. The variables *Post*, *weekend*, *Christmas*, and *Media* were coded as 0 or 1. When testing for the presence of significant direct effects and interactions, the four-level categorical variable *Practice* represented an omnibus test of any possible differences among the four categories, as in a repeated measures ANOVA. To generate change estimates using planned contrasts, *Practice* variable was broken down into three dummy-coded variables, one indicating Observing-thoughts meditation (OTM; 0 or 1), one indicating Loving-kindness meditation (LKM; 0 or 1), and one indicating Body Scan (0 or 1), with Breathing Meditation as the reference group.

Rather than using difference scores, we take a polynomial approach where both “pre-meditation” and “post-meditation” values are included in the dependent variable (differentiated by the predictor *Post*). A difference score implicitly forces the effects of the two variables being differenced to be equal in size but opposite in sign (Edwards, 2001). The polynomial approach estimates “pre-meditation” and “post-meditation” effects simultaneously within one model without making unfounded assumptions about their relationship, and is recommended when estimating change in a regression context (Edwards, 2001).

For the “Warmth” model only, mean daily temperature in the participant’s home post code, mean-centered, was included as an additional covariate and as an interaction term with “Post” in order to ascertain that differences between meditations in perceived warmth were not due to differences in weather across the training modules. Mean daily temperature information for the two study centers was obtained from the Deutscher Wetterdinest Climate Data Center (Deutscher Wetterdinest Climate Data Center, 2014)

We also tested whether the state effects of practice were moderated by a linear effect for the passage of time. As shown in the online supplemental material, this was not consistently the case for any of the variables, and thus the interactions of *time* with *Post* and *Practice* are not included in the final models described here. The equations for the final model available in the supplementary material.

The equations for the within-person model are shown below in Raudenbush and Bryk (2002) notation.

Level 1: $DV = \beta_{0pi} + \beta_{1pi}(Post) + \beta_{2pi}(Media) + \beta_{3pi}(weekend) + \beta_{4pi}(time) + \gamma_{500}(Christmas) + e_{tpi}$

Level 2: $\beta_{0pi} = \beta_{00i} + \gamma_{010}(Body\ Scan) + \gamma_{020}(LKM) + \gamma_{030}(OTM) + u_{0pi}$

$\beta_{1pi} = \beta_{10i} + \gamma_{110}(Body\ Scan) + \gamma_{120}(LKM) + \gamma_{130}(OTM) + u_{1pi}$

$\beta_{2pi} = \beta_{20i} + u_{2pi}$

$\beta_{3pi} = \beta_{30i} + u_{3pi}$

$\beta_{4pi} = \beta_{40i} + u_{4pi}$

Level 3: $\beta_{00i} = \gamma_{000} + \gamma_{001}(age) + \gamma_{002}(gender) + r_{00i}$

$\beta_{10i} = \gamma_{100} + r_{10i}$

$\beta_{20i} = \gamma_{200} + r_{20i}$

$\beta_{30i} = \gamma_{300} + r_{30i}$

$\beta_{40i} = \gamma_{400} + r_{40i}$

Fixed effects: $DV = \gamma_{000} + \gamma_{001}(age) + \gamma_{002}(gender) + \gamma_{010}(Body\ Scan) + \gamma_{020}(LKM) + \gamma_{030}(OTM) + \gamma_{100}(Post) + \gamma_{110}(Body\ Scan)(Post) + \gamma_{120}(LKM)(Post) + \gamma_{130}(OTM)(Post) + \gamma_{200}(Media) + \gamma_{300}(weekend) + \gamma_{400}(time) + \gamma_{500}(Christmas)$

Random effects: $DV = u_{0pi} + u_{1pi}(Post) + u_{2pi}(Media) + u_{3pi}(weekend) + u_{4pi}(time) + r_{00i} + r_{10i}(Post) + r_{20i}(Media) + r_{30i}(weekend) + r_{40i}(time) + e_{tpi}$

For each variable, a significant effect for γ_{110}, the interaction of meditation type (*Practice*) and change from before the meditation (*Post*), indicates that the amount of change in that variable varies by practice. These significant interactions

were explored using planned contrasts of model-derived estimated change for each practice. Here is an example of such a contrast: To compare the magnitude of state change between LKM and OTM, the amount of change in LKM (represented by the interaction of LKM and Post, γ_{120}) is subtracted from the amount of change in OTM (represented by the interaction of OTM and Post, γ_{130}), then divided by a standard error term derived from the mean square error of the variables *Practice, Post*, and their interaction to produce a *t*-value for which a *p*-value can be calculated. Contrasts were computed using the R package *multcomp* (Hothorn, Bretz & Westfall, 2008). If the interaction term was not significant but γ_{100}, the coefficient representing whether there was significant change across practices (*Post*), was significant, individual state change estimates were still computed in order to demonstrate the magnitude of the state change.

Description of final between-person model

Group-mean centered time, whether the measurement took place on a weekend (*weekend*), whether the measurement was done online or using the smartphone app (*Media*), participant gender, and participant age were included as covariates to improve model fit or improve interpretability of results. Further tests of random effects resulted in keeping correlated random effects for intercept, *Post, time, Media,* and *weekend*. When testing for the presence of significant direct effects and interactions, the three-level categorical variable *group* represented an omnibus test of any possible differences among the four categories, as in a repeated measures ANOVA. To generate change estimates using planned contrasts, the *group* variable was broken down into two dummy-coded variables, one indicating TC1 (0 or 1) and one indicating TC2 (0 or 1), with TC3 as the reference group. The equations for the final model available in the supplementary material.

The equations for the between-person model are shown below in Raudenbush and Bryk (2002) notation.

Level 1: $DV = \beta_{0i} + \beta_{1i}(Post) + \beta_{2i}(Media) + \beta_{3i}(weekend) + \beta_{4i}(time) + e_{ti}$

Level 2: $\beta_{0i} = \gamma_{00} + \gamma_{01}(age) + \gamma_{02}(gender) + \gamma_{03}(TC1) + \gamma_{04}(TC2) + u_{0i}$

$$\beta_{1i} = \gamma_{10} + \gamma_{11}(TC1) + \gamma_{12}(TC2) + u_{1i}$$

$$\beta_{2i} = \gamma_{20} + u_{2i}$$

$$\beta_{3i} = \gamma_{30} + u_{3i}$$

$$\beta_{4i} = \gamma_{40} + u_{4i}$$

Fixed effects:

$DV = \gamma_{00} + \gamma_{01}(age) + \gamma_{02}(gender) + \gamma_{03}(TC1) + \gamma_{04}(TC2) + \gamma_{10}(Post) + \gamma_{11}(Post)(TC1) + \gamma_{12}(Post)(TC2) +$
$\gamma_{20}(Media) + \gamma_{30}(weekend) + \gamma_{40}(time)$

Random effects: $DV = u_{0i} + u_{1i}(Post) + u_{2i}(Media) + u_{3i}(weekend) + u_{4i}(time) + e_{tpi}$

The critical coefficient for testing the hypothesis that order of training influences Loving-kindness meditation is γ_{12}, which represents the interaction of change from pre to post and training cohort. If this coefficient was significant, post-hoc

contrasts were fitted to determine which group was driving the difference in effects. In addition, in the absence of an interaction we tested whether γ_{10} , the effect of change from pre to post, was significant. If it was, or if a significant interaction was present, we used contrasts to compute model-derived change scores for each training cohort and to test whether the change was significant.

Network analysis

Because this technique does not presently allow for repeated measures or within-person effects, we used associations between model-derived "average" change scores for each person for each meditation, drawn from the within-person models described above. This also allowed us to calculate correlations among variables that were not measured on the same day, such as the cube of thought measures and the meta-cognition measures. Using the "qgraph" package in R (Epskamp, Cramer, Waldorp, Schmittmann, & Borsboom, 2012), we computed partial correlation matrices for all the change variables (Future, Past, Self, Others, Positive, Negative, Affect, Energy, Warmth, Present, Body Awareness, Thought Distraction, and Thought Awareness) for each of the four meditative practices. We collapsed across TC1 and TC2 to create a larger sample size and thus more stable estimates. Network analysis for TC3 was conducted separately. After computing the significance tests for all correlations, we corrected for multiple testing using the Benjamini and Hochberg (2000) method to decrease the expected proportion of false discoveries among rejected hypotheses. We did not use the more conservative Bonferroni correction as we did not anticipate that all correlations would be significant.

Tests of nested random effects for within-person models

	TC1					TC2				
Random Effect Nesting	df	logLikelihood	Comparison	Likelihood ratio	p-value	df	logLikelihood	Comparison	Likelihood ratio	p-value
**Future**										
1: Participant/Practice	17	-25822				17	-25163			
2: Participant	16	-25907	1 vs 2	170.37	<.0001	16	-25240	1 vs 2	154.65	<.0001
3: None	15	-26614	2 vs 3	1412.88	<.0001	15	-25977	2 vs 3	1473.30	<.0001
**Past**										
1: Participant/Practice	17	-25151				17	-24406			
2: Participant	16	-25195	1 vs 2	86.32	<.0001	16	-24502	1 vs 2	192.33	<.0001
3: None	15	-25999	2 vs 3	1607.97	<.0001	15	-25227	2 vs 3	1449.81	<.0001
**Self**										
1: Participant/Practice	17	-25415				17	-24682			
2: Participant	16	-25537	1 vs 2	242.60	<.0001	16	-24780	1 vs 2	196.93	<.0001
3: None	15	-26426	2 vs 3	1778.36	<.0001	15	-25514	2 vs 3	1467.63	<.0001
**Others**										
1: Participant/Practice	17	-25815				17	-24936			
2: Participant	16	-25919	1 vs 2	207.67	<.0001	16	-25125	1 vs 2	379.56	<.0001
3: None	15	-26430	2 vs 3	1022.79	<.0001	15	-25836	2 vs 3	1421.47	<.0001
**Positive**										
1: Participant/Practice	17	-24957				17	-23964			
2: Participant	16	-25085	1 vs 2	256.61	<.0001	16	-24085	1 vs 2	242.68	<.0001
3: None	15	-26016	2 vs 3	1863.26	<.0001	15	-25207	2 vs 3	2244.24	<.0001
**Negative**										
1: Participant/Practice	17	-24619				17	-23793			
2: Participant	16	-24680	1 vs 2	122.22	<.0001	16	-23855	1 vs 2	122.86	<.0001
3: None	15	-25733	2 vs 3	2104.72	<.0001	15	-25027	2 vs 3	2345.57	<.0001
**Affect**										
1: Participant/Practice	17	-47163				17	-42644			
2: Participant	16	-47507	1 vs 2	689.15	<.0001	16	-43051	1 vs 2	814.07	<.0001
3: None	15	-50861	2 vs 3	6707.89	<.0001	15	-46725	2 vs 3	7346.80	<.0001
**Energy**										

1: Participant/Practice	17	-50406				17	-46190			
2: Participant	16	-51000	1 vs 2	1188.11	<.0001	16	-46817	1 vs 2	1254.24	<.0001
3: None	15	-53942	2 vs 3	5883.91	<.0001	15	-50098	2 vs 3	6561.59	<.0001
Warmth										
1: Participant/Practice	17	-67828				17	-60536			
2: Participant	16	-68763	1 vs 2	1869.89	<.0001	16	-61078	1 vs 2	1083.54	<.0001
3: None	15	-73529	2 vs 3	9532.21	<.0001	15	-65747	2 vs 3	9338.04	<.0001
Present										
1: Participant/Practice	17	-68802				17	-64739			
2: Participant	16	-69558	1 vs 2	1512.45	<.0001	16	-65718	1 vs 2	1958.17	<.0001
3: None	15	-76447	2 vs 3	13776.55	<.0001	15	-71067	2 vs 3	10698.41	<.0001
Body Aware										
1: Participant/Practice	17	-69958				17	-64671			
2: Participant	16	-70847	1 vs 2	1779.16	<.0001	16	-65742	1 vs 2	2141.91	<.0001
3: None	15	-77777	2 vs 3	13860.01	<.0001	15	-72264	2 vs 3	13045.72	<.0001
Distraction										
1: Participant/Practice	17	-23851				17	-21575			
2: Participant	16	-24017	1 vs 2	333.24	<.0001	16	-21685	1 vs 2	218.93	<.0001
3: None	15	-25320	2 vs 3	2605.67	<.0001	15	-23463	2 vs 3	3556.35	<.0001
Thought Aware										
1: Participant/Practice	17	-23753				17	-21887			
2: Participant	16	-23974	1 vs 2	443.32	<.0001	16	-22227	1 vs 2	679.55	<.0001
3: None	15	-26648	2 vs 3	5347.21	<.0001	15	-24186	2 vs 3	3918.34	<.0001

Note: Model 3, "None," represents a regular linear regression model with no random effects. Model 2, "Participant," is fitted with a random intercept for each participant. Model 1, "Participant/Practice," is fitted with a random intercept for each participant within each practice. All models retained the same fixed effects as the final model presented in the manuscript.

Moderation of state change by time

	TC1				TC2			
Name	numDF	denDF	F-value	p-value	numDF	denDF	F-value	p-value
Future								
Practice:Time	3	9772	0.41	.74	3	9855	1.97	.12
Post:Time	1	9772	0.68	.41	1	9855	3.44	.06
Practice:Post:Time	3	9772	4.54	.00	3	9855	4.43	.00
Past								
Practice:Time	3	9769	1.22	.30	3	9854	0.26	.86
Post:Time	1	9769	0.18	.67	1	9854	0.46	.50
Practice:Post:Time	3	9769	0.40	.76	3	9854	2.29	.08
Self								
Practice:Time	3	9766	1.39	.24	3	9854	0.51	.67
Post:Time	1	9766	1.09	.30	1	9854	1.28	.26
Practice:Post:Time	3	9766	0.79	.50	3	9854	2.10	.10
Others								
Practice:Time	3	9765	2.52	.06	3	9854	1.10	.35
Post:Time	1	9765	0.17	.68	1	9854	0.02	.88
Practice:Post:Time	3	9765	4.47	.00	3	9854	2.64	.05
Positive								
Practice:Time	3	9765	2.83	.04	3	9854	0.87	.45
Post:Time	1	9765	7.37	.01	1	9854	2.40	.12
Practice:Post:Time	3	9765	1.67	.17	3	9854	3.68	.01
Negative								
Practice:Time	3	9764	1.43	.23	3	9853	0.57	.64
Post:Time	1	9764	0.74	.39	1	9853	0.04	.85
Practice:Post:Time	3	9764	1.01	.39	3	9853	3.27	.02
Affect								
Practice:Time	3	30416	11.42	.00	3	29504	6.77	.00
Post:Time	1	30416	11.53	.00	1	29504	1.80	.18
Practice:Post:Time	3	30416	3.86	.01	3	29504	0.60	.61
Energy								
Practice:Time	3	30416	3.36	.02	3	29504	1.55	.20
Post:Time	1	30416	21.73	.00	1	29504	3.97	.05
Practice:Post:Time	3	30416	4.11	.01	3	29504	0.62	.60
Warmth								
Practice:Time	3	30409	28.00	.00	3	29498	4.84	.00
Post:Time	1	30409	2.12	.15	1	29498	0.02	.88
Practice:Post:Time	3	30409	2.74	.04	3	29498	1.80	.14
Present								
Practice:Time	3	30404	5.66	.00	3	29498	0.86	.46
Post:Time	1	30404	13.15	.00	1	29498	9.24	.00
Practice:Post:Time	3	30404	5.11	.00	3	29498	5.26	.00

Body Aware								
Practice:Time	3	30406	7.34	.00	3	29502	3.68	.01
Post:Time	1	30406	4.65	.03	1	29502	22.23	.00
Practice:Post:Time	3	30406	1.37	.25	3	29502	2.39	.07
Thought Distraction								
Practice:Time	3	9916	3.81	.01	3	9456	3.62	.01
Post:Time	1	9916	6.88	.01	1	9456	5.24	.02
Practice:Post:Time	3	9916	1.92	.12	3	9456	3.17	.02
Thought Awareness								
Practice:Time	3	9915	2.49	.06	3	9454	0.29	.83
Post:Time	1	9915	0.89	.35	1	9454	4.36	.04
Practice:Post:Time	3	9915	2.87	.03	3	9454	1.09	.35

Note: In cases where both TC1 and TC2 showed a significant effect, the interactions were probed, and in all cases revealed inconsistent effects across training cohorts. As stated in the manuscript, only consistent effects found in both training cohorts 1 and 2 are interpreted.

Significance tests for all predictors for within-person models

	TC1				TC2			
Name	numDF	denDF	F-value	p-value	numDF	denDF	F-value	p-value
Intercept	1	9779	2086.97	.00	1	9862	2345.71	.00
Age	1	75	5.77	.02	1	75	3.47	.07
Gender	1	75	0.43	.51	1	75	0.05	.82
Practice	3	224	31.54	.00	3	222	32.24	.00
Post	1	9779	49.70	.00	1	9862	92.95	.00
Practice:Post	3	9779	27.72	.00	3	9862	21.25	.00
Media	1	9779	17.39	.00	1	9862	0.94	.33
Weekend	1	9779	0.94	.33	1	9862	2.58	.11
Time	1	9779	11.83	.00	1	9862	11.73	.00
Christmas	1	9779	0.08	.78	1	9862	0.63	.43
Intercept	1	9776	980.71	.00	1	9861	1392.24	.00
Age	1	75	1.82	.18	1	75	1.75	.19
Gender	1	75	4.10	.05	1	75	1.60	.21
Practice	3	224	33.52	.00	3	222	52.69	.00
Post	1	9776	16.41	.00	1	9861	11.20	.00
Practice:Post	3	9776	19.77	.00	3	9861	13.49	.00
Media	1	9776	32.55	.00	1	9861	60.75	.00
Weekend	1	9776	0.00	.99	1	9861	0.06	.80
Time	1	9776	4.01	.05	1	9861	9.69	.00
Christmas	1	9776	1.52	.22	1	9861	2.34	.13
Intercept	1	9773	1961.08	.00	1	9861	2944.99	.00
Age	1	75	6.56	.01	1	75	0.12	.73
Gender	1	75	0.35	.56	1	75	0.62	.43
Practice	3	224	12.02	.00	3	222	11.25	.00
Post	1	9773	0.17	.68	1	9861	0.55	.46
Practice:Post	3	9773	0.53	.66	3	9861	0.83	.48
Media	1	9773	1.18	.28	1	9861	0.53	.47
Weekend	1	9773	3.40	.07	1	9861	4.26	.04
Time	1	9773	1.70	.19	1	9861	2.51	.11
Christmas	1	9773	0.06	.81	1	9861	0.07	.79
Intercept	1	9772	2643.06	.00	1	9861	2476.01	.00
Age	1	75	0.51	.48	1	75	9.66	.00
Gender	1	75	0.75	.39	1	75	5.07	.03
Practice	3	224	23.56	.00	3	222	51.75	.00
Post	1	9772	34.57	.00	1	9861	69.11	.00
Practice:Post	3	9772	49.38	.00	3	9861	43.45	.00
Media	1	9772	33.51	.00	1	9861	17.21	.00
Weekend	1	9772	0.00	.95	1	9861	1.99	.16
Time	1	9772	0.43	.51	1	9861	0.21	.65
Christmas	1	9772	0.05	.82	1	9861	4.09	.04
Intercept	1	9772	2787.41	.00	1	9861	2710.94	.00
Age	1	75	0.97	.33	1	75	5.48	.02
Gender	1	75	0.02	.89	1	75	0.20	.66
Practice	3	224	7.83	.00	3	222	13.79	.00
Post	1	9772	0.36	.55	1	9861	1.31	.25
Practice:Post	3	9772	10.51	.00	3	9861	13.19	.00

Practice	3	224	7.83	.00	3	222	13.79	.00
Post	1	9772	0.36	.55	1	9861	1.31	.25
Practice:Post	3	9772	10.51	.00	3	9861	13.19	.00
Media	1	9772	5.61	.02	1	9861	4.14	.04
Weekend	1	9772	0.93	.34	1	9861	1.32	.25
Time	1	9772	8.48	.00	1	9861	2.39	.12
Christmas	1	9772	9.69	.00	1	9861	0.54	.46
Intercept	1	9771	562.24	.00	1	9860	612.53	.00
Age	1	75	4.18	.04	1	75	3.98	.05
Gender	1	75	9.30	.00	1	75	2.18	.14
Practice	3	224	14.69	.00	3	222	18.45	.00
Post	1	9771	130.66	.00	1	9860	56.68	.00
Practice:Post	3	9771	9.58	.00	3	9860	16.13	.00
Media	1	9771	8.39	.00	1	9860	18.34	.00
Weekend	1	9771	1.96	.16	1	9860	0.12	.72
Time	1	9771	0.07	.79	1	9860	1.06	.30
Christmas	1	9771	7.89	.00	1	9860	0.88	.35
Intercept	1	30423	5066.80	.00	1	29511	3564.74	.00
Age	1	75	0.33	.57	1	75	3.78	.06
Gender	1	75	0.43	.51	1	75	0.83	.36
Practice	3	225	8.59	.00	3	223	11.42	.00
Post	1	30423	113.71	.00	1	29511	123.70	.00
Practice:Post	3	30423	7.61	.00	3	29511	4.33	.00
Media	1	30423	25.25	.00	1	29511	27.26	.00
Weekend	1	30423	8.42	.00	1	29511	0.00	.96
Time	1	30423	5.16	.02	1	29511	0.87	.35
Christmas	1	30423	14.30	.00	1	29511	21.91	.00
Intercept	1	30423	3270.21	.00	1	29511	3418.87	.00
Age	1	75	0.26	.61	1	75	3.38	.07
Gender	1	75	0.17	.69	1	75	0.01	.93
Practice	3	225	10.62	.00	3	223	10.44	.00
Post	1	30423	51.54	.00	1	29511	73.48	.00
Practice:Post	3	30423	24.32	.00	3	29511	4.11	.01
Media	1	30423	1.60	.21	1	29511	11.86	.00
Weekend	1	30423	6.17	.01	1	29511	8.47	.00
Time	1	30423	1.51	.22	1	29511	0.19	.66
Christmas	1	30423	4.58	.03	1	29511	3.89	.05
Intercept	1	30414	4557.17	.00	1	29503	4694.81	.00
Age	1	75	1.89	.17	1	75	2.63	.11
Gender	1	75	1.06	.31	1	75	0.66	.42
Practice	3	225	22.88	.00	3	223	7.13	.00
Post	1	30414	27.50	.00	1	29503	46.06	.00
Practice:Post	3	30414	14.45	.00	3	29503	5.03	.00
Media	1	30414	30.13	.00	1	29503	69.45	.00
Weekend	1	30414	3.17	.07	1	29503	0.01	.91
Time	1	30414	7.65	.01	1	29503	1.64	.20
Christmas	1	30414	2.36	.12	1	29503	2.66	.10
[illegible]	[illegible]	[illegible]	[illegible]	[illegible]	[illegible]	[illegible]	[illegible]	[illegible]

Temperature	1	30414	888.62	.00	1	29503	91.41	.00
Post:Temperature	1	30414	26.37	.00	1	29503	10.44	.00
Intercept	1	30411	2233.56	.00	1	29505	2957.19	.00
Age	1	75	1.37	.25	1	75	12.32	.00
Gender	1	75	1.66	.20	1	75	2.39	.13
Practice	3	225	2.52	.06	3	223	2.42	.07
Post	1	30411	135.34	.00	1	29505	122.16	.00
Practice:Post	3	30411	0.99	.40	3	29505	0.93	.43
Media	1	30411	33.29	.00	1	29505	36.11	.00
Weekend	1	30411	5.06	.02	1	29505	0.05	.83
Time	1	30411	0.82	.36	1	29505	10.10	.00
Christmas	1	30411	11.07	.00	1	29505	2.75	.10
Intercept	1	30413	2206.22	.00	1	29509	2396.73	.00
Age	1	75	0.77	.38	1	75	10.85	.00
Gender	1	75	4.07	.05	1	75	4.53	.04
Practice	3	225	5.23	.00	3	223	5.34	.00
Post	1	30413	108.57	.00	1	29509	121.54	.00
Practice:Post	3	30413	14.31	.00	3	29509	10.61	.00
Media	1	30413	25.31	.00	1	29509	31.07	.00
Weekend	1	30413	6.44	.01	1	29509	0.01	.92
Time	1	30413	1.45	.23	1	29509	6.75	.01
Christmas	1	30413	3.20	.07	1	29509	11.50	.00
Intercept	1	9923	1487.75	.00	1	9463	1061.62	.00
Age	1	75	0.56	.46	1	75	0.00	.95
Gender	1	75	0.23	.64	1	75	0.02	.89
Practice	3	223	6.56	.00	3	222	4.89	.00
Post	1	9923	74.22	.00	1	9463	80.53	.00
Practice:Post	3	9923	9.89	.00	3	9463	2.48	.06
Media	1	9923	9.71	.00	1	9463	5.12	.02
Weekend	1	9923	0.85	.36	1	9463	0.05	.83
Time	1	9923	0.00	.96	1	9463	5.47	.02
Christmas	1	9923	0.25	.62	1	9463	2.20	.14
Intercept	1	9922	1487.81	.00	1	9461	1597.29	.00
Age	1	75	1.18	.28	1	75	1.21	.27
Gender	1	75	0.00	.98	1	75	0.52	.47
Practice	3	223	10.72	.00	3	222	9.36	.00
Post	1	9922	9.35	.00	1	9461	7.92	.00
Practice:Post	3	9922	23.91	.00	3	9461	14.09	.00
Media	1	9922	2.24	.13	1	9461	4.96	.03
Weekend	1	9922	0.06	.80	1	9461	0.42	.52
Time	1	9922	5.21	.02	1	9461	6.48	.01
Christmas	1	9922	2.87	.09	1	9461	0.19	.66

Significance tests for all predictors for between-person model

	numDF	denDF	F-value	p-value
Intercept	1	7043	4778.26	.00
Training Cohort	2	217	3.62	.03
Post	1	7043	105.59	.00
Time	1	7043	11.20	.00
Media	1	7043	1.56	.21
Gender	1	217	1.36	.24
Age	1	217	3.72	.05
Weekend	1	7043	1.45	.23
Training Cohort: Post	2	7043	1.28	.28
Intercept	1	7041	2475.76	.00
Training Cohort	2	217	0.76	.47
Post	1	7041	31.56	.00
Time	1	7041	1.56	.21
Media	1	7041	39.10	.00
Gender	1	217	0.37	.54
Age	1	217	1.34	.25
Weekend	1	7041	0.09	.77
Training Cohort: Post	2	7041	1.02	.36
Intercept	1	7039	5195.50	.00
Training Cohort	2	217	1.71	.18
Post	1	7039	2.87	.09
Time	1	7039	1.61	.20
Media	1	7039	4.23	.04
Gender	1	217	0.97	.32
Age	1	217	1.21	.27
Weekend	1	7039	0.18	.67
Training Cohort: Post	2	7039	0.41	.67
Intercept	1	7038	7662.33	.00
Training Cohort	2	217	0.47	.63
Post	1	7038	9.97	.00
Time	1	7038	0.23	.63
Media	1	7038	8.12	.00
Gender	1	217	1.48	.23
Age	1	217	0.73	.39
Weekend	1	7038	4.82	.03
Training Cohort: Post	2	7038	0.30	.74
Intercept	1	7038	6393.19	.00
Training Cohort	2	217	2.66	.07
Post	1	7038	41.97	.00
Time	1	7038	0.00	.98
Media	1	7038	7.11	.01
Gender	1	217	0.11	.74
Age	1	217	1.52	.22
Weekend	1	7038	2.09	.15
Training Cohort: Post	2	7038	0.68	.51
Intercept	1	7038	1391.13	.00
Training Cohort	2	217	3.25	.04
Post	1	7038	132.00	.00
Time	1	7038	0.02	.88
Media	1	7038	8.14	.00
Gender	1	217	1.80	.17

Gender	1	217	1.89	.17
Age	1	217	3.02	.08
Weekend	1	7038	0.05	.83
Training Cohort: Post	2	7038	2.82	.06
Intercept	1	20950	9739.77	.00
Training Cohort	2	219	0.36	.70
Post	1	20950	235.05	.00
Time	1	20950	3.94	.05
Media	1	20950	10.10	.00
Gender	1	219	0.94	.33
Age	1	219	0.48	.49
Weekend	1	20950	0.18	.67
Training Cohort: Post	2	20950	4.43	.01
Intercept	1	20950	7503.06	.00
Training Cohort	2	219	1.33	.27
Post	1	20950	115.81	.00
Time	1	20950	7.17	.01
Media	1	20950	0.59	.44
Gender	1	219	3.65	.06
Age	1	219	4.85	.03
Weekend	1	20950	14.19	.00
Training Cohort: Post	2	20950	1.00	.37
Intercept	1	20937	8296.79	.00
Training Cohort	2	219	0.41	.67
Post	1	20937	171.46	.00
Time	1	20937	3.94	.05
Media	1	20937	51.18	.00
Gender	1	219	1.52	.22
Age	1	219	0.51	.47
Weekend	1	20937	1.20	.27
Temperature	1	20937	18.62	.00
Training Cohort: Post	2	20937	17.10	.00
Post: Temperature	1	20937	1.32	.25
Intercept	1	20942	4915.84	.00
Training Cohort	2	219	0.64	.53
Post	1	20942	164.71	.00
Time	1	20942	12.43	.00
Media	1	20942	34.11	.00
Gender	1	219	8.99	.00
Age	1	219	2.33	.13
Weekend	1	20942	0.16	.69
Training Cohort: Post	2	20942	1.39	.25
Intercept	1	20942	4431.23	.00
Training Cohort	2	219	0.79	.46
Post	1	20942	154.79	.00
Time	1	20942	22.42	.00
Media	1	20942	28.15	.00
Gender	1	219	12.50	.00
Age	1	219	3.43	.07
Weekend	1	20942	2.55	.11
Training Cohort: Post	2	20942	0.34	.72
Intercept	1	6644	2833.12	.00
Training Cohort	[illegible]	[illegible]	[illegible]	[illegible]

Training Cohort	2	217	7.51	.00
Post	1	6644	133.01	.00
Time	1	6644	1.73	.19
Media	1	6644	10.99	.00
Gender	1	217	0.38	.54
Age	1	217	2.46	.12
Weekend	1	6644	0.12	.73
Training Cohort: Post	2	6644	0.30	.74
Intercept	1	6643	3298.86	.00
Training Cohort	2	217	0.37	.69
Post	1	6643	13.15	.00
Time	1	6643	9.63	.00
Media	1	6643	2.57	.11
Gender	1	217	8.97	.00
Age	1	217	0.01	.91
Weekend	1	6643	3.10	.08
Training Cohort: Post	2	6643	0.28	.76

Within-person model results without covariates

$DV = \beta_{0pi} + \beta_{1pi}(Post) + e_{tpi}$

Level 2: $\beta_{0pi} = \beta_{00i} + \gamma_{010}(Body\ Scan) + \gamma_{020}(LKM) + \gamma_{030}(OTM) + u_{0pi}$

$\beta_{1pi} = \beta_{10i} + \gamma_{110}(Body\ Scan) + \gamma_{120}(LKM) + \gamma_{130}(OTM) + u_{1pi}$

Level 3: $\beta_{00i} = \gamma_{000} + r_{00i}$

$\beta_{10i} = \gamma_{100} + r_{10i}$

Fixed effects: $DV = \gamma_{000} + \gamma_{010}(Body\ Scan) + \gamma_{020}(LKM) + \gamma_{030}(OTM) + \gamma_{100}(Post) + \gamma_{110}(Body\ Scan)(Post) + \gamma_{120}(LKM)(Post) + \gamma_{130}(OTM)(Post)$

Random effects: $DV = u_{0pi} + u_{1pi}(Post) + r_{00i} + r_{10i}(Post) + e_{tpi}$

	Training Cohort 1					Training Cohort 2				
	Breathing	Body Scan	Loving-kindness	Observing-thoughts	γ_{110} p-value	Breathing	Body Scan	Loving-kindness	Observing-thoughts	γ_{110} p-value
Future	**-1.18**[a]	**-2.32**	**-1.4**[a]	0.18	0	**-1.51**[a]	**-2.47**[b]	**-1.97**[ab]	-0.31	0
Past	**-0.41**	**-0.91**[a]	**-0.91**[a]	**0.69**	0	-0.21	**-0.85**[a]	**-0.7**[a]	**0.47**	0
Self	-0.13[a]	0.08[a]	-0.02[a]	0.19[a]	0.6	-0.01[a]	0.12[a]	0.4[a]	0.2[a]	0.5
Others	**-1.75**	**-2.88**	0.18[a]	-0.36[a]	0	**-1.99**	**-2.61**	**0.61**	-0.51	0
Positive	**-0.43**[a]	**-0.63**[a]	**0.58**	-0.16[a]	0	-0.25[a]	-0.16[a]	**1.12**	0.02[a]	0
Negative	**-0.78**[a]	**-1.17**[b]	**-1.32**[b]	**-0.38**[a]	0	**-0.64**	**-1.13**	**-1.58**	-0.24	0
Affect	**0.44**[a]	**0.52**[a]	**0.38**[a]	**0.29**	0	**0.49**[a]	**0.64**	**0.51**[a]	**0.51**[a]	0.01
Energy	**0.38**[a]	**0.78**	**0.42**[a]	**0.44**[a]	0	**0.51**[a]	**0.74**[b]	**0.57**[ac]	**0.67**[bc]	0.01
Warmth	**0.33**[a]	0.11[a]	**0.73**[b]	**0.75**[b]	0	**0.46**[ab]	**0.27**[a]	**0.67**[b]	**0.66**[b]	0
Present	**1.72**[a]	**1.81**[a]	**1.56**[a]	**1.62**[a]	0.34	**1.7**[a]	**1.93**[a]	**1.75**[a]	**1.86**[a]	0.45
Body Aware	**1.5**[a]	**2.35**	**1.39**[a]	**1.7**[a]	0	**1.39**[a]	**2.33**	**1.45**[a]	**1.72**[a]	0
Distraction	**-1.04**[a]	**-2.06**	**-1.45**[b]	**-1.27**[ab]	0	**-1.42**[a]	**-1.83**[b]	**-1.66**[ab]	**-1.49**[ab]	0.1
Thought Aware	**0.84**[a]	-0.23	**0.51**[a]	**1.43**	0	**0.56**[a]	-0.02	**0.69**[a]	**1.25**	0

Effect of season on magnitude of state change in training

The magnitude of state changes in training for Breathing meditation and Body Scan do not differ between TC1 and TC2, despite each cohort being trained in a different season.

	Breathing meditation				Body Scan			
Interaction of Cohort and State Change	numDF	denDF	F-value	p-value	numDF	denDF	F-value	p-value
Future	1	5615	1.03	.31	1	5109	0.17	.68
Past	1	5613	0.67	.41	1	5109	0.09	.76
Self	1	5611	0.20	.66	1	5109	0.01	.93
Others	1	5611	0.65	.42	1	5109	0.44	.51
Positive	1	5611	0.31	.58	1	5109	1.68	.20
Negative	1	5611	0.58	.45	1	5109	0.28	.59
Affect	1	16914	1.08	.30	1	15879	1.77	.18
Energy	1	16914	2.78	.10	1	15879	0.27	.61
Warmth	1	16913	0.58	.45	1	15875	0.65	.42
Present	1	16909	0.00	.98	1	15873	0.79	.37
Body Aware	1	16912	0.02	.89	1	15876	0.02	.89
Distraction	1	5266	1.73	.19	1	5439	0.19	.66
Thought Aware	1	5266	0.61	.43	1	5439	0.24	.63

References

Benjamini, Y., & Hochberg, Y. (2000). On the adaptive control of the false discovery fate in multiple testing with independent statistics. *Journal of Educational and Behavioral Statistics, 25*(1), 60-83. doi:Doi 10.2307/1165312

Edwards, J. R. (2001). Ten difference score myths. *Organizational Research Methods*, *4*(3), 265-287.

Epskamp, S., Cramer, A. O. J., Waldorp, L. J., Schmittmann, V. D., & Borsboom, D. (2012). qgraph: Network visualizations of relationships in psychometric data. *Journal of Statistical Software, 48*(4), 1-18.

Hothorn, T., Bretz, F. & Westfall, P. (2008). Simultaneous inference in general parametric models. *Biometrical Journal, 50*(3), 246-363.

Raudenbush, S. W., & Bryk, A. S. (2002). *Hierarchical linear models : applications and data analysis methods* (2nd ed.). Thousand Oaks: Sage Publications.
